# Enhancing antioxidant activity of rice protein hydrolysates through glycosylation modification

**DOI:** 10.3389/fnut.2025.1616272

**Published:** 2025-07-11

**Authors:** Zhimin Zhang, Xinjian Bi, Liangjie Hu, Ying Wu, Wenting Hu, Han Wang, Xinyue Zheng, Hao Wu, Daichen Mu, Li Wen, Qingming Huang

**Affiliations:** ^1^School of Food Science and Bioengineering, Hunan Provincial Key Laboratory of Cytochemistry, Changsha University of Science and Technology, Changsha, China; ^2^Hunan Zhunong Rice Industry Ltd Co., Yiyang, China

**Keywords:** rice protease hydrolysate, Maillard reaction, zebrafish, antioxidant activity, glycosylation

## Abstract

**Objective:**

This work sought to improve the potential use of rice protein by examining the antioxidant activity of glycosylated rice protein hydrolysates (RPH).

**Methods:**

RPH were produced via the enzymatic breakdown of rice protein powder utilizing trypsin. Then, using the Maillard reaction, these hydrolysates were glycosylated with three functional monosaccharides to create RPH-fructose (RPH-F), RPH-xylose (RPH-X), and RPH-arabinose (RPH-A). The antioxidant capabilities of glycosylated derivatives were assessed *in vitro* by measuring Fe^2+^ chelating ability and their ability to neutralize several radicals, including hydroxyl (•OH), superoxide anion (O_2_•^−^), 2,2-diphenyl-1-picrylhydrazyl radical (DPPH•), and 2,2′-azinobis (3-ethylbenzothiazoline-6-sulfonate) radical (ABTS•^+^). A zebrafish *in vivo* model was utilized to investigate oxidative damage, analyzing the distribution of reactive oxygen species (ROS) through fluorescence staining and evaluating oxidative stress by quantifying malondialdehyde (MDA) levels and the activity of antioxidant enzymes such as catalase (CAT) and total superoxide dismutase (T-SOD). Following glycosylation, DPPH• clearance by RPH-X increased by 12.75% (6 mg/mL), and ROS inhibition by RPH-A in the zebrafish model reached 84.78%.

**Conclusion:**

Glycosylation enhanced the antioxidant capabilities of rice protein hydrolysate, indicating its potential as a functional dietary component with antioxidant efficacy.

## 1 Introduction

Oxidative stress primarily arises from an imbalance between oxidative and antioxidant mechanisms in the body. This imbalance leads to oxidation and the excessive production of ROS, including superoxide anions and hydroxyl radicals (1). These ROS damage cellular macromolecules, accelerating the aging process and leading to functional decreases in cells and tissues, as well as impairing physiological health (2). Therefore, the elimination of excess reactive oxygen species and the suppression of oxidative reactions are crucial goals in the creation of antioxidant products.

Altering glycosylation in diverse ways may modify bioactive peptides’ structure and physicochemical properties, enhancing their biological activity. Three principal ways are utilized for glycosylation: enzymatic approach, non-covalent linkage, and Maillard reaction-based method. Transglutaminase (TGase) is the most often utilized enzyme in glycosylation processes. ﻿Song et al. used the TGase method to glycosylate ﻿soybean isolate protein (SPI) digest and found that the method significantly improved the *in vitro* antioxidant capacity of SPI (3). Nonetheless, its intricate manufacture and preparation method and low biocatalyst stability restrict its applicability (4). Non-covalent linkage methods entail the covalent bonding to particular amino groups of proteins or peptides, while weaker physical interactions facilitate the attachment of peptides to polysaccharides (5). Nevertheless, the majority of these compounds or derivatives frequently demonstrate instability. ﻿Zeng et al. compared various common glycosylation methods and reviewed the progress of glycosylation of casein as an example, making predictions on the limitations and future directions (6). The Maillard reaction is a non-enzymatic glycosylation process involving the condensation of carbonyl and ammonium groups between free amino groups of proteins and carbonyl groups of reducing sugars (7). This process significantly enhances the antioxidant capabilities of oxidized peptides and offers advantages such as simplicity (8), low toxicity, and high efficiency, rendering it a preferred glycosylation method (9).

Functional monosaccharides are monosaccharides exhibiting particular biological functions (e.g., antioxidant, anti-inflammatory). This work involved the glycosylation of RPH with arabinose, xylose, and fructose. Previous studies have indicated that these monosaccharides may enhance protein products’ antioxidant and functional attributes. Arabinose glycosylation effectively decreased fat oxidation (10). Wang ﻿et al. found that the antioxidant capacity of whey protein and xylose glycosylation products was significantly increased (11). Fructose glycosylation diminished the sensitivity of wheat gluten proteins (12). This study utilized the glycosylation of rice protein hydrolysate with certain functional monosaccharides to elucidate their connection with antioxidant capabilities. While the antioxidant improvement of RPH by modifications like xylose has been documented, the structural interactions among various monosaccharides (e.g., xylose, fructose, and arabinose) and their synergistic mechanisms both *in vitro* and *in vivo* remain unclear.

This work involved the production of three different glycosylation products of RPH using the Maillard reaction and assessed the effect of glycosylation modification on external antioxidant ability. A zebrafish model was created to evaluate the *in vivo* antioxidant properties, with reactive oxygen species distribution identified via fluorescent staining. This study establishes a theoretical and technical framework for the processing and production of functional glycosylated RPH, thereby enhancing the industrial applicability and economic worth of rice protein. Moreover, as a sustainable and eco-friendly initiative, it diminishes trial-and-error expenses and curtails resource wastage, enhancing ecologically responsible research methodologies.

## 2 Materials and methods

### 2.1 Experimental materials

Rice protein powder, with a crude protein content of 80%, was obtained from Xinyang Mufan Biotechnology Co. Trypsin, derived from bovine pancreas (1:250), was supplied by Beijing Bailing Wei Technology Co. Fructose, xylose, and arabinose (98%) were purchased from Shanghai McLean Biochemical Technology Co. Ferrozine (97%) and 1,10-phenanthroline (o-diazaphene, 99%) were acquired from Shanghai Macklin Biochemical Technology Co. and Sinopharm Chemical Reagent Co., respectively. DPPH• (﻿97%) was sourced from Teichai (Shanghai) Chemical Industry Development Co., and ABTS•^+^ (﻿98%) came from Shanghai Aladdin Biochemical Technology Co. Glutathione (GSH, 99%) and ROS Measurement Kits (chemiluminescence method), along with T-SOD kits, were provided by Beijing Bailing Wei Technology Co. and Nanjing Jianjian Bioengineering Institute, respectively. The bicinchoninic acid (BCA) kit, CAT kit, and MDA kit were supplied by Beijing Solepol Technology Co. Other chemical reagents, of analytical purity, were obtained from Sinopharm Chemical Reagent Co.

### 2.2 Instruments and equipment

An electronic balance (model AX224ZH) was used from OHAUS Instruments (Changzhou) Co. A medical centrifuge (model TG16-WS) was provided by Changsha Hi-Tech Industrial Development Zone Xiangyi Centrifuge Instrument Co. The magnetic stirring water bath (model HCJ-2E) and the magnetic stirrer (model 8S-1) were supplied by Changzhou Enpei Instrument Manufacturing Co. and Changzhou Guozhi Instrument Manufacturing Co., respectively. The freeze-dryer (model LGJ-25C) was from Sihuan Frui Keji Science and Technology Development Co. The pH meter was provided by METTLER TOLEDO INSTRUMENTS (SHANGHAI) CO. An optical absorption full wavelength enzyme labeling instrument (ReadMax 1900) was used from Shanghai Sempervision Biotechnology Co. The particle size analyzer Dynamic Light Scattering (DLS) was a product of Brookhaven Instruments, United States. The Ultraviolet (UV) spectrophotometer (model UV1800) was from Shimadzu International Trading (Shanghai) Co. A Fourier Transform Infrared Spectrometer (model NicoletiS10) was utilized, provided by Shanghai Zequan Instrument Co.

### 2.3 Preparation of RPH

Modified from previously documented procedures (13, 14), 67 g of rice protein powder were dissolved in 1000 mL of deionized water to form a 1 g/15 mL solution. The solution was stirred at room temperature for 2 h, the pH was adjusted to 8, and then heated in a water bath at 50°C for 15 min. Trypsin was added at an enzyme-to-substrate ratio of 1:100, and the enzymatic reaction was maintained at 50°C for 60 min with a stable pH of 8. After the reaction, the mixture was heated to 95°C for 15 min to deactivate the enzyme, cooled rapidly in an ice bath, and the pH was adjusted to neutral. Following centrifugation (3,500 rpm, 10 min), the supernatant was lyophilized and reserved. The degree of hydrolysis (DH) was determined by the pH-stat method (15), calculating DH according to Equation (1).

(1) $$\mathrm{DH}=\frac{h}{h_{\mathrm{tot}}}\times 100\%=B\times N_{b}\frac{1}{\alpha }\times \frac{1}{M_{p}}\frac{1}{h_{\mathrm{tot}}}\times 100\%$$

In the formula:

B, volume of NaOH consumed (mL); N_b_, concentration of NaOH (mol/L); *α*, dissociation degree of α-NH_3_^+^ at pH = 8 and 50°C under enzymatic hydrolysis conditions is 0.885; M_p_, total protein content in the substrate (g); h_tot_, Theoretical millimoles of peptide bonds per gram of rice protein (7.40 meq/g).

### 2.4 Preparation of glycosylated rice protein hydrolysates

Following the method described in the literature (16) with slight modifications, 2.0 g of sugar and 1.0 g of rice protein digest were weighed, dissolved in 200 mL deionized water, stirred thoroughly, and the pH was set to 7.0. The mixture was reacted in a magnetic stirrer water bath at 80°C for 4 h to produce RPH-F, RPH-X, and RPH-A. Subsequently, it was cooled in an ice bath to room temperature and the samples were dialyzed at low temperature for 24 h. The dialysate was freeze-dried for use.

### 2.5 Structural characterization of glycosylation products

#### 2.5.1 Particle size determination

Following the method described in the literature (16, 17) with slight modifications. To examine the impact of glycosylation modification on the conformation of RPH, the average hydrodynamic particle sizes of the samples were assessed using DLS, which operates on the principle of Brownian motion, calculating diffusion coefficients and inferring size distributions by measuring the fluctuation rate of light scattered by the particles in solution. Take 400 μL of dialysate from enzymatic and glycosylated samples respectively, dilute 10 times, and detect particle size using an analyzer. Equilibrate samples for 10 min and scan each three times for the average particle size.

#### 2.5.2 Determination of grafting degree

Following the method described in the literature (16, 18) with slight modifications. The decrease in free amino acid (FAA) levels, indicative of the glycosylation process, was quantified using O-Phthalaldehyde (OPA). For the analysis, 200 μL aliquots of solutions (RPH-F, RPH-X, RPH-A) were transferred to test tubes. Each sample was combined with 4 mL of OPA reagent and incubated at 35°C for 2 min within a water bath. Subsequently, absorbance at 340 nm was recorded. A control sample of 200 μL RPH was subjected to the same experimental conditions. The degree of grafting (DG) indicates the decrease of free amino groups in glycosylation reactions, with higher DG values indicating enhanced covalent binding effectiveness of sugar molecules to proteins. This work employed the OPA method to ascertain DG, with the objective of identifying the best monosaccharide type (e.g., xylose DG = 16.38%) for further functional examination.

To prepare the OPA reagent, weigh 40 mg of OPA and dissolve it in 1 mL of methanol. Subsequently, add 2.50 mL of a 20% SDS solution, 25 mL of a 0.10 mol/L borax solution, and 100 μL of *β*-mercaptoethanol in that order. Finally, dilute the mixture to a total volume of 50 mL with distilled water.

The degree of grafting (DG) was calculated using Equation (2).

(2) $$\mathrm{DG}=\frac{\left(A_{0}-A_{1}\right)}{A_{0}}\times 100\%$$

In the formula:

A_0_, Absorbance value of the blank sample at 340 nm; A_1_, Absorbance value of the sample at 340 nm.

#### 2.5.3 Fourier transform infrared absorption spectra

Following a method adjusted from (19, 20), four freeze-dried samples (RPH, RPH-F, RPH-X, RPH-A) were mixed and ground with potassium bromide powder at a specific mass ratio. The mixture was pressed into a uniform, transparent flake and analyzed using a Fourier Transform Infrared (FTIR) Spectrometer, scanning from 4,000 cm^−1^- 400 cm^−1^ at a resolution of 2 cm^−1^.

#### 2.5.4 Ultraviolet spectroscopy

Using the method described in reference (16) with minor modifications, RPH, RPH-F, RPH-X, and RPH-A were each dispersed in a phosphate buffer solution (pH 7.0, 50 mmol/L) at a concentration of 0.50 mg/mL. Absorbance scans were performed using a UV–visible spectrophotometer across the wavelength range of 200–600 nm.

### 2.6 *In vitro* antioxidant activity assay

#### 2.6.1 Fe^2+^ chelating capacity

Adapting the method from literature (21) with slight modifications, 0.5 mL of sample solutions (mass concentrations: 6, 3, 1.5, 0.75, and 0.375 mg/mL) were taken into 5 mL centrifugal tubes. Then, 3.20 mL of distilled water and 0.10 mL of 2 mmol/L FeCl_2_ solution were added successively, shaken well, and left for 3 min. Next, 2 mL of 5 mmol/L phenanthroline solution was added, and the reaction occurred at 25°C for 10 min. Absorbance was measured at 562 nm with an enzyme counter. Deionized water served as the blank control and glutathione as the positive control in three parallel experiments. The Fe^2+^ chelating capacity of the samples was calculated according to Equation (3).

(3) $$P=\frac{\left(A_{0}-A_{s}\right)}{A_{0}}\times 100\%$$

In the formula:

P, Fe^2+^ chelating capacity; A_0_, Absorbance of the blank group measured at 562 nm; A_S_, Absorbance of the experimental group measured at 562 nm.

#### 2.6.2 Hydroxyl radical scavenging capacity

Referring to the method in the literature (22) with slight modifications, the reagents were added according to the combinations in the (Table 1), where the concentrations of the sample solution to be tested were 6, 3, 1.5, 0.75, and 0.375 mg/mL in that order.

**Table 1:** Addition of hydroxyl radical regent (mL).

Solution name	Experimental group	Control	Blank group
o-Diazophene solution (1.5 mmol/L)	1.0	1.0	1.0
Phosphate buffer solution (pH 7.4)	2.0	2.0	2.0
Distilled water	–	1.0	1.0
Ferrous sulfate solution (1.5 mmol/L)	1.0	1.0	1.0
0.02% hydrogen peroxide solution	1.0	1.0	–
Distilled water	–	–	1.0
Sample solution to be measured	1.0	–	–

The solutions were thoroughly mixed, and reactions were conducted for 60 min at 37°C in a water bath. Absorbance at 536 nm was measured, with the procedure repeated in triplicate. The scavenging activity of the samples against hydroxyl radicals was quantified according to Equation (4).

(4) $$P=\frac{A_{2}-A_{1}}{A_{0}-A_{1}}\times 100\%$$

In the formula:

P, hydroxyl radical scavenging rate; A_0_, absorbance measured in the blank group; A_1_, Absorbance measured for the control; A_2_, absorbance measured by the experimental group.

#### 2.6.3 Superoxide anion radical scavenging capacity

Following modifications from literature (22), 1.0 mL of sample solutions was placed into a 10 mL centrifuge tube. Each tube received 3.00 mL of 50 mmol/L Tris–HCl buffer (pH 8.2), reacted at 25°C for 20 min, followed by the addition of 3.00 mL of 7 mmol/L pyrogallol solution, and reacted for another 5 min. The reaction was terminated with 1 mL of concentrated HCl, and absorbance was measured at 325 nm. This procedure was repeated in triplicate, and the superoxide anion radical scavenging rate was determined according to Equation (5).

(5) $$P=\frac{A_{0}-(A_{1}-A_{2})}{A_{0}}\times 100\%$$

In the formula:

P, superoxide anion radical scavenging rate; A_0_, absorbance at 325 nm of deionized water instead of sample solution; A_1_, absorbance of the sample solution at 325 nm; A_2_, absorbance at 325 nm of the sample solution without pyrogallol added.

#### 2.6.4 DPPH• scavenging rate

Following the method described in the literature (16, 23) with slight modifications. In the experimental system, 3.0 mL of DPPH• solution was added with 1.0 mL of the sample solution for the experimental group, while anhydrous ethanol substituted the DPPH• solution in the control group, and the sample solvent replaced the sample in the blank group. The concentrations of the sample solution under investigation were 6, 3, 1.5, 0.75, and 0.375 mg/mL, respectively. Sample solutions were formulated with differing concentrations. The components were properly mixed and permitted to react for 60 min in darkness prior to measuring the absorbance at 517 nm. Deionized water functioned as the zero calibration standard, while glutathione served as the positive control. The method was repeated thrice, and the scavenging efficacy against DPPH• was determined using Equation (6).

(6) $$P=(1-\frac{A_{s}-A_{c}}{A_{b}})\times 100\%$$

In the formula:

P, DPPH• scavenging rate; A_s_, absorbance measured by the experimental group; A_c_, absorbance measured for the control; A_b_, absorbance measured in the blank group.

#### 2.6.5 ABTS•^+^ scavenging rate

Following the method described in the literature (16, 24) with slight modifications. In the experimental group, 3.6 mL of ABTS•^+^ solution was combined with 0.4 mL of sample solution. The control group substituted the samples with solvent, with the concentrations of the test solution being 6, 3, 1.5, 0.75, and 0.375 mg/mL, respectively, mixed thoroughly, and allowed to react for 5 min in darkness. Absorbance was subsequently quantified at 734 nm, utilizing deionized water for zero calibration and glutathione as the positive control. The method was conducted in triplicate, and the scavenging activity against ABTS•^+^ was determined using Equation (7).

(7) $$P=(\frac{A_{b}-A_{s}}{A_{b}})\times 100\%$$

In the formula:

P, ABTS•^+^ scavenging rate; A_b_, absorbance measured in the blank group; A_s_, absorbance measured by the experimental group.

### 2.7 *In vivo* antioxidant activity assay

#### 2.7.1 Zebrafish culture and collection of zygotic embryos

Wild-type AB strain zebrafish were employed in this study. Feeding of zebrafish was conducted separately by sex in the breeding tank, with one spoonful of shelled Toyon shrimp eggs provided bi-daily at 09:00 and 17:00. The temperature was regulated at 28°C ± 0.5°C, and a light–dark cycle of 14 h of light and 10 h of darkness was maintained. Zebrafish embryos were obtained by the natural mating spawning technique. At 18:30 on the eve of breeding, male and female zebrafish were positioned in a 1:1 ratio on either side of the breeding tank, divided by a baffle, and maintained in darkness overnight. At 08:30 the following day, the barriers were dismantled and the lighting system was activated to facilitate zebrafish mating and spawning. Zebrafish embryos were gathered and maintained in an incubator at 28.5°C.

#### 2.7.2 Assessment of the provided dosage

Juvenile fish (3 day post fertilization, dpf) were placed randomly in 6-well plates (4 mL solution volume/well, 30 fish/well). The test group was treated with RPH-A solutions at concentrations of 50.0, 100.0, 200.0, 400.0, and 800.0 μg/mL. Model and normal control groups were established. Except for the normal group, all others were exposed to 300 μM hydrogen peroxide (H_2_O_2_) to induce oxidative damage in zebrafish. After 24 h, the number of deceased fish in each group was recorded. The highest RPH-A concentration without causing fish death or morphological abnormalities was determined as the maximum toxic concentration (MTC).

#### 2.7.3 *In vivo* ROS detection and antioxidant evaluation in zebrafish

Slightly modified with reference to (25). Juvenile zebrafish (3 dpf) were divided randomly into four groups: Control (blank control group), Model (300 μM H_2_O_2_), RPH group (200 μg/mL RPH + 300 μM H_2_O_2_), and RPH-A group (200 μg/mL RPH-A + 300 μM H_2_O_2_), with three replicates in each group. Samples were incubated with a 40 μg/mL fluorescent probe for 20 min for ROS labeling, followed by photography using an *in vivo* fluorescence microscope to assess fluorescence intensity.

#### 2.7.4 Determination of peroxidase activity in zebrafish

Juvenile zebrafish (3 dpf, normal development) were divided randomly into four groups: Control (blank control group), Model (300 μM H_2_O_2_), GSH group (20 μg/mL glutathione + 300 μM H_2_O_2_), and RPH-A group (200 μg/mL RPH-A + 300 μM H_2_O_2_), with three replicates for each. The treatments were conducted over 4 days using the protocols provided with the MDA, CAT, and T-SOD kits. The MDA concentration, CAT activity, and T-SOD activity were measured separately using a UV spectrophotometer.

### 2.8 Statistical analysis

Data were analyzed using SPSS software, with results presented as “mean ± standard deviation (SD).” A one-way analysis of variance (ANOVA) was utilized to assess differences among groups, considering *p* < 0.05 as statistically significant.

## 3 Results and discussion

### 3.1 Degree of hydrolysis of enzymes

The hydrolysis degree of rice protein via trypsin in this experiment was 5.98%. According to the optimization experiments of ﻿Yang et al. (26), the hydrolysis degree of rice protein by trypsin hydrolysis can reach 54.0% under optimum conditions. A strong correlation exists between the extent of hydrolysis of enzymatic hydrolysate and factors such as substrate concentration, starting pH, enzyme quantity, reaction temperature, and hydrolysis time. Moreover, Ma ﻿et al. (27) discovered that excessive hydrolysis compromises the structure of enhanced rice protein, resulting in diminished heat resistance. Additionally, varying degrees of hydrolysis through different restriction enzymes also influenced the *in vitro* antioxidant activity of RPH (28).

### 3.2 Particle size determination

**Figure 1:**
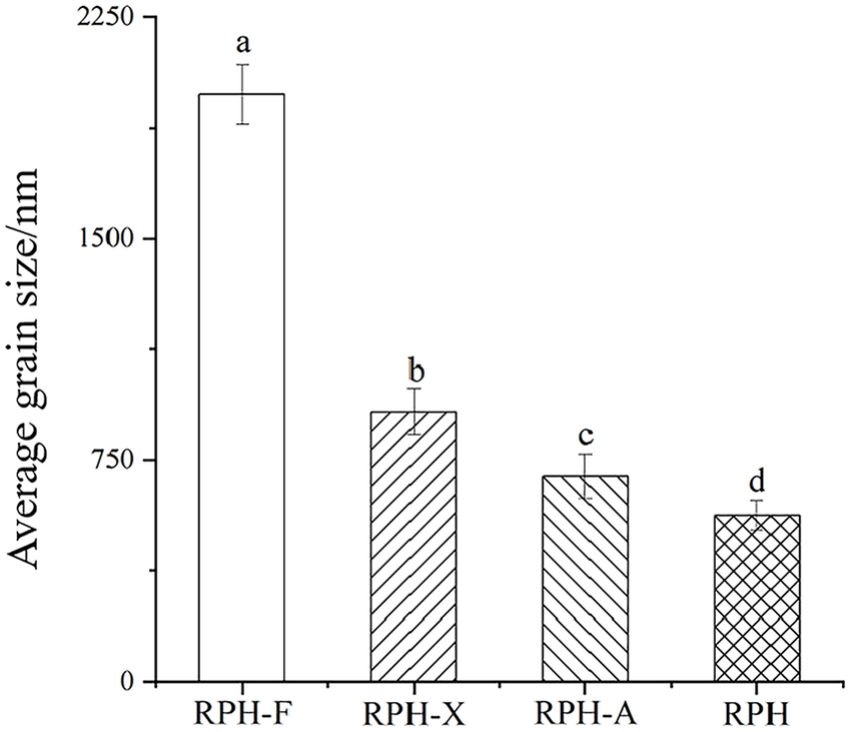
Effects of different glycosylation reactions on the average particle size of RPH.

Glycosylation increased the average particle size of all products: specifically, RPH-F was enlarged by over 3.5 times relative to RPH, RPH-X by over 1.6 times, and RPH-A by up to 1.2 times compared to RPH (﻿Figure 1). These changes suggest that modified RPHs adopt a looser structural organization. Ma ﻿et al. noted that the addition of monosaccharide molecules alters the conformation of protein, leading to an increase in particle size (29). Variations in particle size among glycosylated products might stem from the differing accessibility of functional sugars to various protein sites (30). The larger the molecular weight of the sugar, the greater the obstruction to protein compactness. Given fructose’s larger molecular weight, RPH-F exhibited the greatest increase in particle size under identical glycosylation condition. Xylose and arabinose exhibit variations in their glycosylation sites on proteins; arabinose preferentially binds to the hydroxyl or amine groups of proteins, while xylose may be associated through distinct glycosidic linkages. This may lead to RPH-X forming more expansive, less compact structures during glycosylation, whereas RPH-A produces denser formations (31).

### 3.3 Grafting degree determination

**Figure 2:**
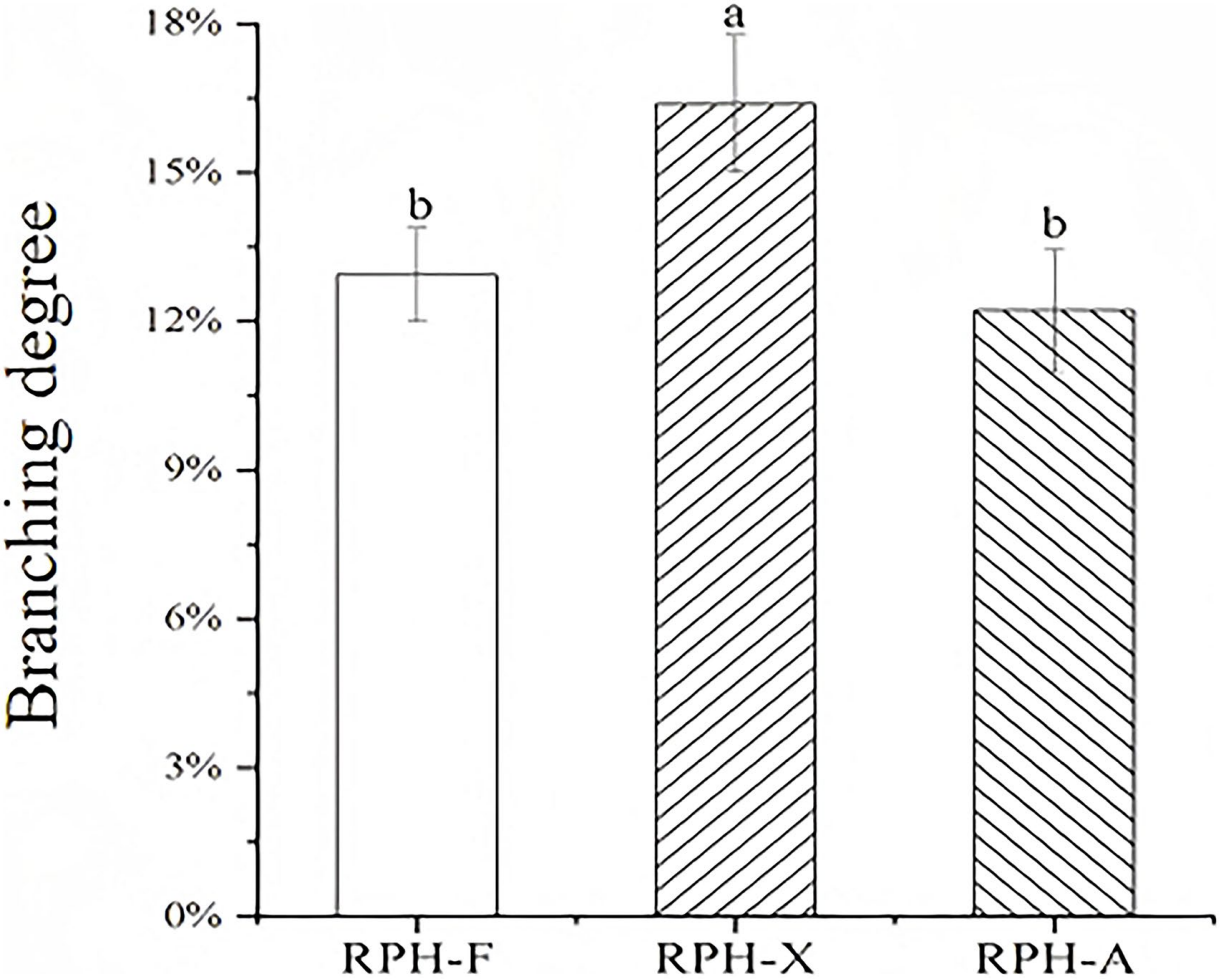
Grafting degrees of different glycosylation reaction products.

The grafting degree for RPH-X, derived from the reaction between rice protein digest and xylose, reached a maximum of 16.38% under identical experimental conditions (Figure 2), consistent with previous studies (16). The physicochemical and functional characteristics of grafted rice protein glycosylation products were comparable. A high grafting degree suggests a reduction in FAA content in the glycosylated product, indicating that certain amino acids are covalently bonded to sugars (32). The binding capacity of RPH to the three sugars was ranked as follows: xylose > fructose > arabinose. This hierarchy may be attributed to the chemical structures of the sugars and their affinity for proteins. The relatively lower grafting efficiency of fructose and arabinose could be due to structural differences that reduce their binding capacity (33). The aldehyde group of xylose demonstrates higher reactivity compared to the ketone group of arabinose, whereas the cyclic structure of arabinose reduces its accessibility to the peptide chain, resulting in a difference in grafting degree (RPH-X: 16.38% vs. RPH-A: 8.72%) (34). Xylose had the greatest glycosylation efficiency, presumably due to the elevated reactivity of its aldehyde group, which promotes enhanced interactions with proteins.

### 3.4 Fourier infrared absorption spectra

**Figure 3:**
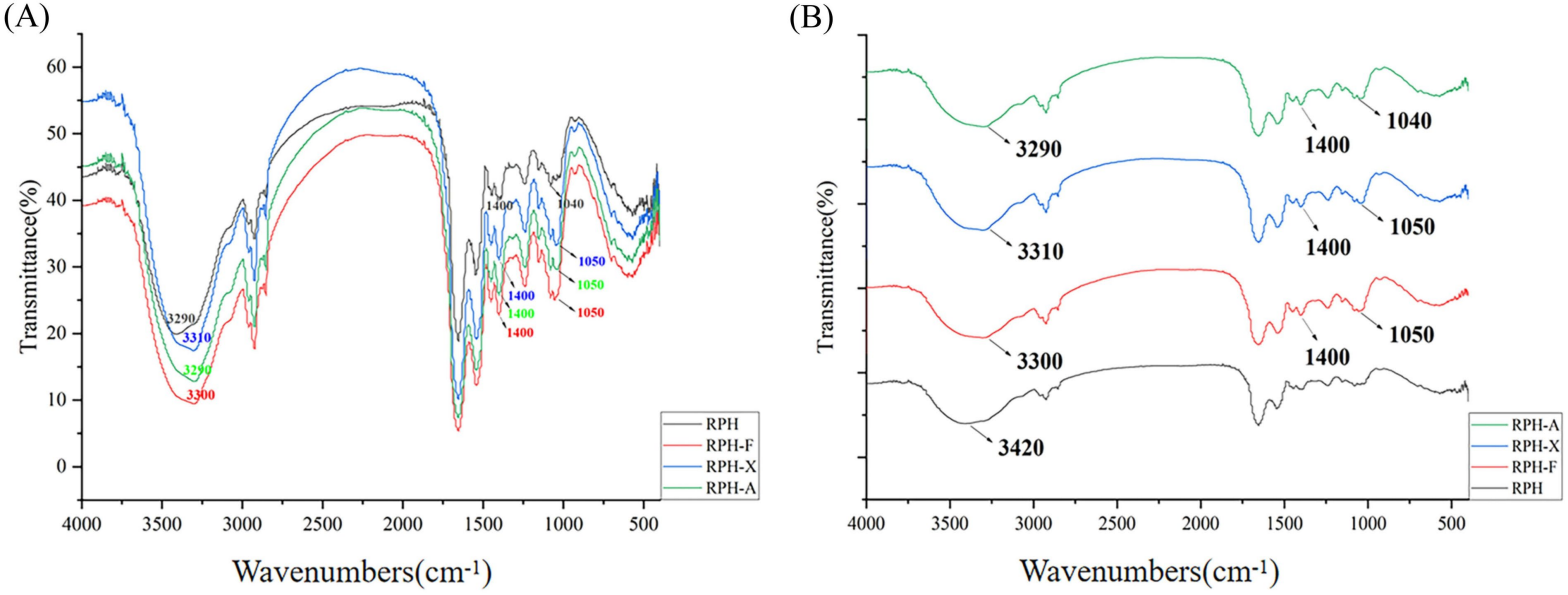
**(A)** Infrared spectra of RPH and its glycosylated products. **(B)** Infrared spectral expansion of RPH and its glycosylated products.

FTIR spectroscopy is employed for protein structural analysis, as it detects characteristic absorption peaks in the mid-infrared region, reflecting changes in the peptide chain structure. The wavelength range of 3,500–3,000 cm^−1^ corresponds to the characteristic absorption peak of -OH groups (Figure 3). Compared to RPH, the three glycosylation products exhibited enhanced absorption intensity (decreased transmittance) and a slight blue shift in the absorption peaks at 3500–3000 cm^−1^. This shift indicates the stretching vibration of the -OH group and the formation of new -OH bonds, suggesting the occurrence of a glycosylation reaction. Since sugar molecules are rich in -OH groups, their attachment to RPH via covalent bonds leads to an increased presence of -OH groups.

Moreover, the highly polar nature of -OH groups enable them to establish hydrogen bonds, which accounts for the wider absorption peaks ranging from 3,650–3,200 cm^−1^ in the conjugates (35). Absorption peaks found between 1,000–1,070 cm^−1^ typically reflect the C-O-C stretching vibrations and the presence of glycan rings within sugar molecules (36). At 1050 cm^−1^, a notable increase in absorption peak intensity was evident across all three glycosylated products, suggesting that the sugar molecules prompted vibrations in the protein side chains as a result of the glycosylation process. In addition, each of the three glycosylation products exhibited a distinct absorption peak between 1,380–1,410 cm^−1^. This peak, attributable to the -CN stretching vibration, arises from carbonyl-ammonia condensation (37) and indicates an augmentation in glycosidic bond formation.

### 3.5 Ultraviolet spectroscopy

**Figure 4:**
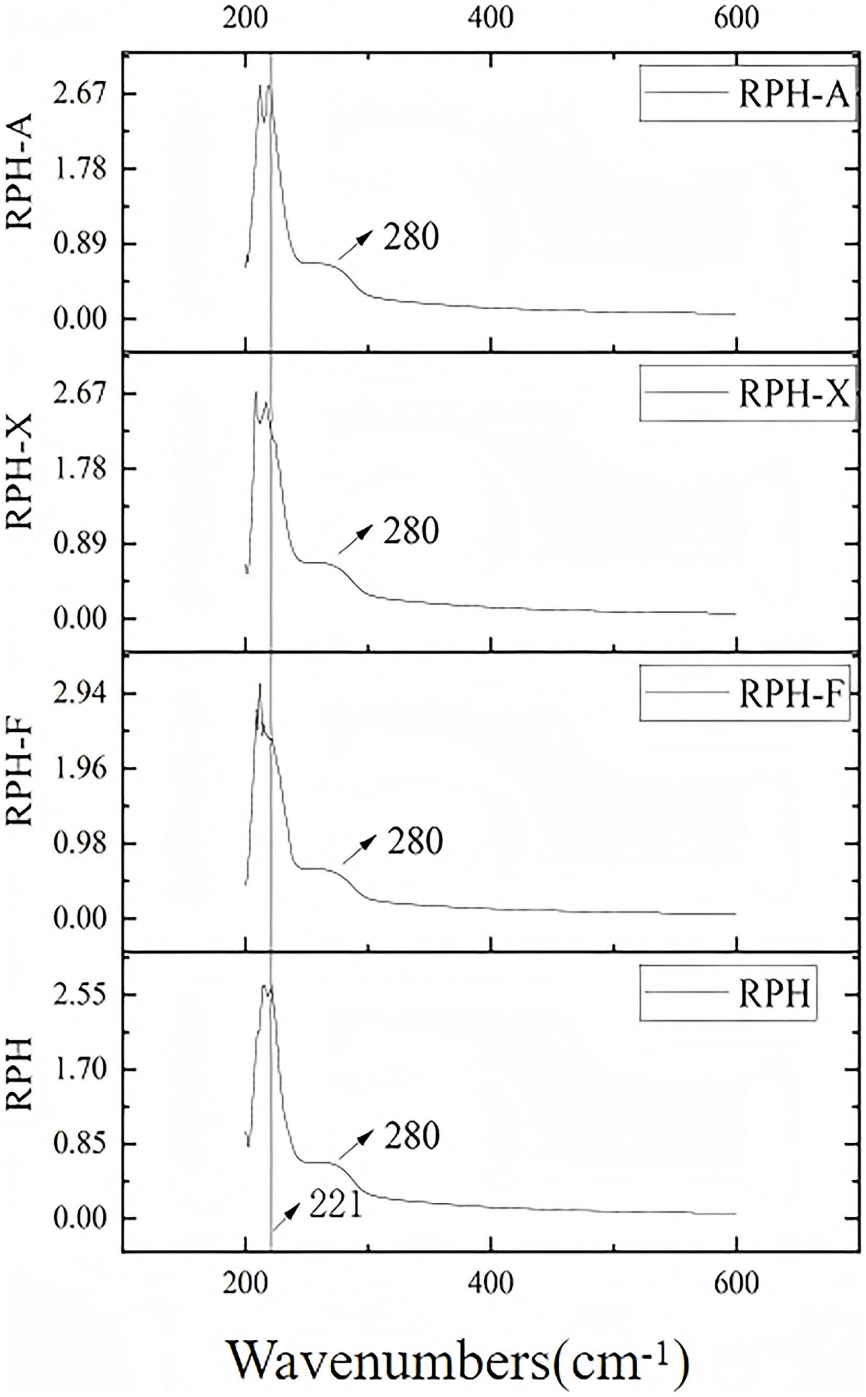
UV spectra of RPH and its glycosylated products.

Aromatic amino acids, such as tyrosine, tryptophan, and phenylalanine, present in RPH (38), absorb ultraviolet light at specific wavelengths, particularly at 225 nm and 280 nm (Figure 4). The glycosylation product showed a slight blue shift in UV absorption, peaking at 221 nm. This shift suggests that glycosylation may alter the protein’s three-dimensional structure, especially the conformation of peptide chains. Likely due to the Maillard reaction, which forms covalent bonds between amino acids and sugars, these alterations modify molecular interactions. Consequently, structural changes expose certain hydrophobic amino acid side chains, increasing their interaction with the environment. This exposure might explain the enhanced peak in UV absorption (9).

### 3.6 *In vitro* antioxidant activity assay

#### 3.6.1 Fe^2+^ chelating capacity

**Figure 5:**
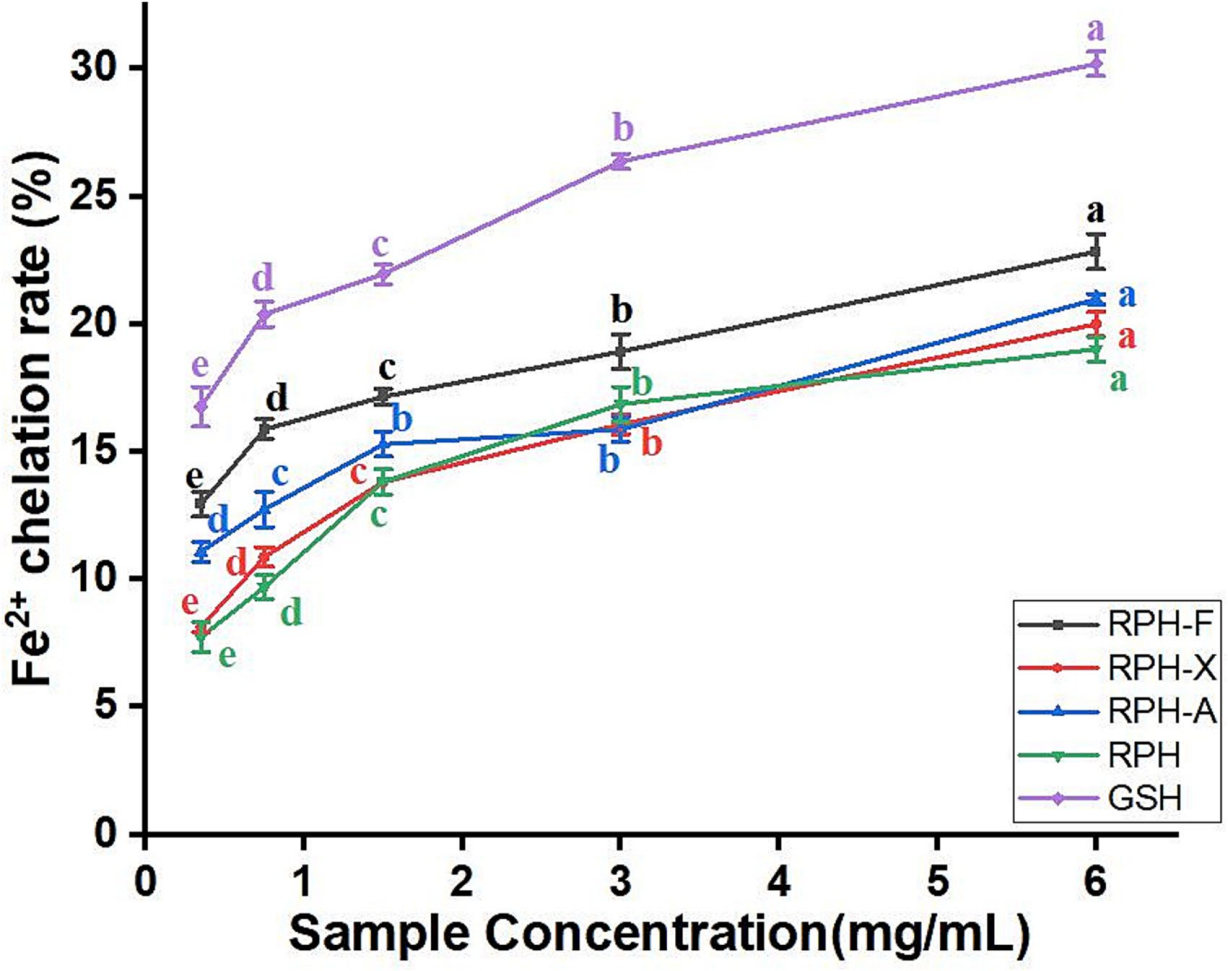
Fe^2+^chelating ability of different glycosylation products.

The Fe^2+^ chelating ability of both enzymatic hydrolysates and glycosylation products increased with rising concentrations, from 0.375 to 6 mg/mL. A significant rise of 6.23% in Fe^2+^ chelating capacity was noted at a 0.75 mg/mL concentration of RPH-F (Figure 5), indicating superior antioxidant activity in RPH-F compared to other glycosylated variants. This enhancement is likely due to the unique structure of RPH-F formed during glycosylation and the molecular structure of xylose, which enhances its Fe^2+^ binding. The exposure of carbonyl groups on amino acid residues, which possess strong coordination capabilities, allows for more effective Fe^2+^ complex formation (39). By chelating Fe^2+^, glycosylated RPH may reduce oxidative damage by preventing the generation of free radicals (20). Furthermore, glycosylation alters the protein’s spatial conformation, exposing more hydrophilic and hydrophobic regions, which improves its chelating capability and antioxidant properties.

#### 3.6.2 Hydroxyl radical scavenging capacity

**Figure 6:**
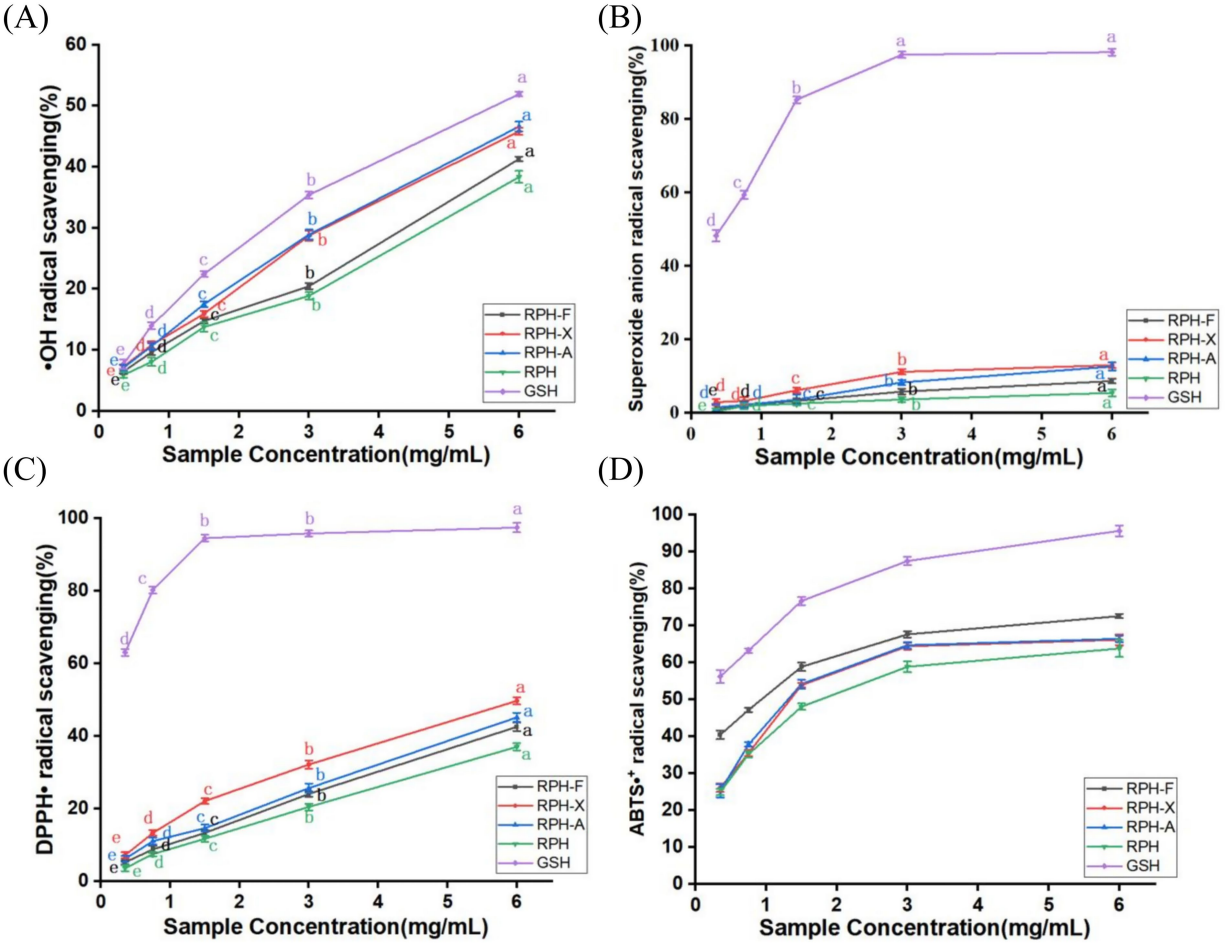
**(A)** •OH radical scavenging rate of different glycosylation products. **(B)** Superoxide anion radical scavenging rate of different glycosylation products. **(C)** Scavenging rate of DPPH*•* by different glycosylation products. **(D)** Scavenging rate of ABTS•^+^ by different glycosylation products.

Hydroxyl radicals, capable of causing extensive damage to human cells and leading to lipid peroxidation (23), are more effectively scavenged by glycosylated products. The scavenging activity of these products increased with concentration. Among them, RPH-F showed the least effective •OH scavenging capability (Figure 6A). This effect is attributable to the glycosylation products acting as chelating agents that bind with metal ions such as Fe^2+^, inhibiting hydroxyl radical production and consequently reducing oxidation. The variation in antioxidant properties among glycosylated products is linked to the type of glycosyl groups and the degree of modification (40). Zhang et al. investigated Maillard reaction products of derived from soy protein isolate with L-arabinose and D-galactose, demonstrating that glycosylation with L-arabinose resulted in superior antioxidant activity, primarily due to its higherdegree of glycation and more substantial effects on protein conformation (41). Similarly, Xiao et al. reported that solid-state fermentation significantly enhanced the free radical scavenging capacity of buckwheat by releasing bound phenolic compounds through microbial hydrolytic enzymes (42). Although native rice protein typically exhibits limited antioxidant activity, its simple structure, minimal branching, and high content of reactive amino acids such as lysine and arginine, render it particularly suitable for non-enzymatic glycosylation, enabling the formation of uniform and stable glycosylation products (33).

#### 3.6.3 Superoxide anion radical scavenging capacity

Superoxide anion radicals, which can evolve into highly reactive hydroxyl radicals (43), were tested for scavenging potential. Even at a maximum concentration of 6 mg/mL, the scavenging capacity of RPH did not exceed 15% (Figure 6B). However, glycosylated samples displayed a significant improvement, with RPH-F showing the most notable enhancement. This indicates that glycosylation effectively enhances the antioxidant properties of RPH. This is consistent with the findings of Wang et al., who demonstrated that glycosylation of yak casein with glucose via the Maillard reaction significantly enhanced its superoxide anion scavenging capacity, and this effect increased proportionally with higher sugar concentrations (44). Additionally, Limsuwanmanee et al. reported a similar observation in a Maillard reaction system involving aquatic by-products and various monosaccharides, finding that glycosylation substantially improved the products’ O_2_•^−^ scavenging activity with smaller monosaccharides (such as fructose and glucose) exhibiting more pronounced effects (45). As superoxide anion radicals are precursors to highly reactive substances, their scavenging capacity closely mirrored the trend observed for hydroxyl radicals (Figures 6A,B). Compared to RPH, glycosylation enhanced the superoxide anion radical scavenging ability, likely due to the polyhydroxyl structure of the sugars and the antioxidant-active amino acid residues. These modifications allow the glycosylated products to act as effective hydrogen donors, neutralizing superoxide anion radicals (28). Furthermore, glycosylation modifications have been shown to improve the solubility and stability of proteins, the introduction of hydrophilic hydroxyl groups from sugars increases surface hydrophilicity, thereby enhancing water solubility, enhancing their ability to interact with reactive substances (40).

#### 3.6.4 DPPH• scavenging capacity

DPPH• is a stable nitrogen-centered radical, absorbing maximally at 517 nm. The purple ethanol solution of DPPH•, and its concentration, relate linearly to absorbance. The antioxidant capacity of a substance can thus be determined by measuring its ability to scavenge DPPH• at 517 nm (46). Both RPH and glycosylation products showed increased scavenging activity as the concentration increased, with glycosylation products demonstrating higher DPPH• scavenging rates than the hydrolysates. In this study, we observed a 12.75% increase in the DPPH• scavenging rate of RPH-X (Figure 6C), aligning with the trend of enhanced DPPH• activity reported by Chen et al. in fermented soybeans (47). Wang et al. reported that yak casein glycosylated via the Maillard reaction exhibited significantly greater DPPH• scavenging capacity compared to its native form, with efficacy strongly correlated with the extent of glycation (44). Similarly, Dai et al. demonstrated that enzymatic hydrolysis of wheat gluten with subsequent Maillard glycosylation significantly enhanced its DPPH• scavenging ability, achieving up to 71.71% clearance under optimal conditions (48). Compared with proteins such as casein and gluten, rice protein inherently possesses relatively low antioxidant activity, possibly due to its compact structure and limited hydrophobic group exposure. However, the protein’s favorable chemical reactivity allows substantial improvement of antioxidant properties through glycation. Previous studies have demonstrated that the Maillard reaction markedly enhances DPPH• scavenging capacity by increasing protein hydrophilicity, revealing reactive functional sites, and introducing hydrogen-donating groups (33).

#### 3.6.5 ABTS•^+^ scavenging capacity

ABTS is oxidized to form ABTS•^+^, imparting a stable blue-green color with maximum absorption at 734 nm. Antioxidant components react with ABTS•^+^, leading to discoloration of the reaction system, and the scavenging capacity is calculated (16). The ABTS•^+^ scavenging capacity of glycosylated rice proteolytic digests progressively increased with increasing concentration within a specific range. Furthermore, the ABTS•^+^ scavenging capacity of glycosylated rice protein digests exceeded 50%. At a concentration of 3 mg/mL, the scavenging rates of RPH-X, RPH-A, and RPH-F were 60.7, 61.2, and 63.5%, respectively, (Figure 6D). The sequence of ABTS•^+^ scavenging potency was RPH-F > RPH-A > RPH-X. This enhanced activity of RPH and its derivatives might be due to the formation of more potent free radical-scavenging compounds during glycosylation, potentially similar to black essence-like structures (39). Chen et al. found that solid-state fermentation significantly increased the contents of total phenolics, total flavonoids, and aglycone isoflavones in soybeans, thereby enhancing their scavenging abilities against free radicals such as ABTS•^+^ and DPPH• (49). These findings further highlight the crucial role of structural modification in improving free radical scavenging capacity. Additionally, the ABTS•^+^ scavenging rate showed a positive correlation with sample concentration, a pattern consistent with the other antioxidant activity-concentration relationships observed.

### 3.7 Evaluation of *in vivo* antioxidant effect

#### 3.7.1 Examination of administered dose

As the tolerated concentration of RPH-A in zebrafish is unknown, a preliminary experiment was conducted. For each concentration gradient, 90 zebrafish were selected with three parallel groups established for each concentration. This gradient was used to determine the maximum tolerated concentration (MTC) of the enzyme hydrolysates in zebrafish, providing essential reference data for evaluating their antioxidant efficacy.

As indicated in Table 2 under the experimental conditions, when zebrafish were exposed to RPH-A at concentrations below 200.0 μg/mL, both the experimental and control groups exhibited no deaths or morphological abnormalities. However, as the concentration of RPH-A increased, a gradual increase in zebrafish mortality was observed, suggesting that the toxicity of RPH-A is positively correlated with its concentration. The MTC of RPH-A for zebrafish was established at 200.0 μg/mL, consistent with previous findings (50).

**Table 2:** Experimental result of maximum detectable concentration of zebrafish (*n* = 30).

Groups	RPH-A sample concentration/(μg/mL)	Zebrafish mortality	Zebrafish mortality rate/%	Phenotype
Normal control group	–	0	0	No apparent abnormality
Model Control group	–	0	0	No apparent abnormality
Test group	50.0	0	0	Similar status to Modeled controls
	100.0	0	0	Similar status to Modeled controls
	200.0	0	0	Similar status to Modeled controls
	400.0	5	17	–
	800.0	30	100	–

#### 3.7.2 Detection of ROS and evaluation of antioxidant effects in zebrafish

Hydrogen peroxide, a known oxidant, can significantly elevate ROS levels when organisms are exposed to external stressors, exceeding their natural antioxidant defenses and causing oxidative stress. This imbalance may result in considerable cellular damage (51). A common method for detecting ROS in zebrafish involves the use of fluorescent probes, such as dichlorodihydrofluorescein diacetate (DCFH-DA), which crosses cell membranes and is oxidized to fluorescent compounds upon ROS exposure. The antioxidant effect of RPH-A in zebrafish is assessed by measuring fluorescence intensity. The fluorescence intensity results, after continuous exposure for 96 h, are presented below:

Fluorescence intensity is presented in ImageJ as a pixel value, grayscale value, or optical density value. A pixel is the basic unit of an image, and its numerical value represents the intensity.

**Figure 7:**
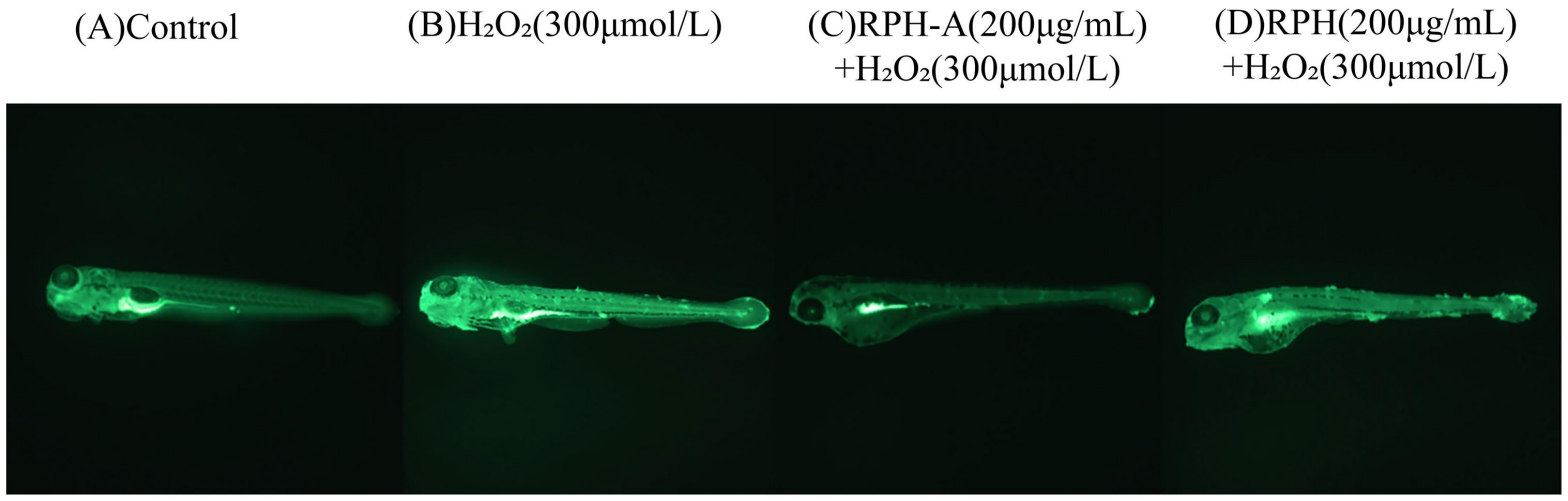
Typical diagrams of fluorescence intensity in zebrafish.

As depicted in Figure 7 and Table 3 fluorescence intensity in the head, heart, and trunk regions of zebrafish larvae in the model group was significantly higher, confirming successful establishment of the oxidative damage model and elevated ROS levels in the zebrafish. These observations align with findings from Luo et al. (52). The fluorescence intensities in the RPH-A and RPH groups were lower, indicating that both RPH and its glycosylated form, RPH-A, effectively reduced ROS levels in zebrafish. The enhanced bioactivity observed might result from glycation-induced structural modifications that alter cell membrane permeability (53), thereby facilitating deeper tissue penetration. Moreover, glycation-induced conformational stabilization protects critical antioxidant amino acid residues (e.g., Tyr and Trp) from degradation or oxidation, consequently improving their stability and durability (54).

**Table 3:** Evaluation experiment results on the antioxidant efficacy of glycosylated enzymatic hydrolysates.

Groups	Fluorescence intensity (pixels, mean±SE)	Relative fluorescence intensity	Antioxidant effect/%
Control	581,453 ± 13684.66	0.015	–
Model	36,507,700 ± 1447285.79	1.000	–
RPH	25,229,703 ± 1476000.38***	0.691	31.39***
RPH-A	6,045,490 ± 432304****	0.170	84.78****

Comparison with model control group, ***p < 0.001, ****p < 0.0001.

### 3.8 ﻿Effects of RPH-A on peroxidase activity in zebrafish

**Figure 8:**
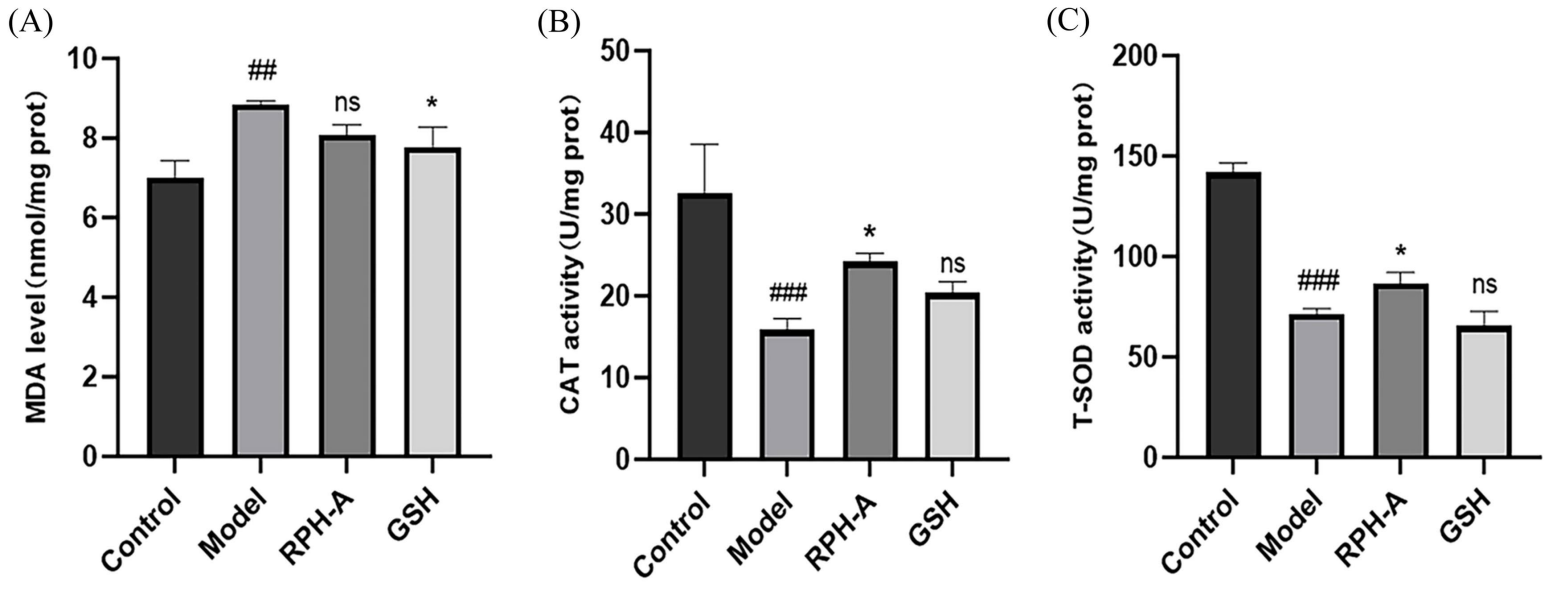
Zebrafish body MDA levels and peroxidase activity. **(A)** MDA levels. **(B)** CAT activity. **(C)** T-SOD activity. The * sign indicates a significant difference, *p < 0.05 The # sign represents a significant difference in the model control group, # (*p* < 0.05), ## (*p* < 0.01), ### (*p* < 0.001), ns indicates no significant difference (*p* > 0.05).

MDA levels, indicative of lipid peroxidation and thus oxidative stress, were significantly elevated in the model group (Figure 8A; *p* < 0.01), validating the model’s effectiveness (55). Conversely, MDA concentrations were lower in the GSH-treated group, underscoring its protective role against lipid peroxidation.

CAT, prevalent in animals, plants, microbes, and cultured cells, is the predominant H_2_O_2_ scavenging enzyme and plays a crucial part in the reactive oxygen species scavenging mechanism. In the model group, CAT activity was significantly decreased (*p* < 0.001), suggesting an impairment of the antioxidant defense mechanism. Conversely, in the RPH-A treated group, CAT activity was notably higher (*p* < 0.05; Figure 8B), indicating that RPH-A plays a role in enhancing antioxidant defenses. Additionally, CAT levels in the GSH-treated group exceeded those in the model group, supporting findings from earlier studies (56).

T-SOD is a metalloenzyme prevalent in living organisms. It serves as a crucial oxygen radical scavenger that catalyzes the disproportionation of superoxide anion to produce H_2_O_2_ and O_2_ (50). T-SOD functions as both a superoxide anion scavenging enzyme and a principal H_2_O_2_-producing enzyme, significantly contributing to the biological antioxidant system. Regarding T-SOD activity, it was substantially reduced in the model group (*p* < 0.001). However, T-SOD activity was elevated in the RPH-A treated group (*p* < 0.05), demonstrating RPH-A’s protective role in zebrafish, as illustrated in Figure 8C. Comparatively, T-SOD levels in the model group and the GSH-treated group showed no significant differences (*p* > 0.05) (57).

## 4 Conclusion

This study employed a combined enzymatic hydrolysis and glycosylation modification approach to enhance the antioxidant capacity of RPH, which were subsequently evaluated through *in vitro* and *in vivo* assays. Glycosylation significantly enhanced the ability to scavenge free radicals; at a concentration of 6 mg/mL, the DPPH radical scavenging rate of RPH-X was 12.75% greater than that of unglycosylated RPH. Moreover, glycosylated hydrolysates exhibited robust ROS scavenging activity, with an *in vivo* antioxidant efficacy reaching 84.78%. Additionally, glycosylated hydrolysates markedly elevated the activities of antioxidant enzymes, such as CAT and T-SOD. These findings suggest that glycosylated RPH exhibit substantial antioxidant potential in both *in vitro* and *in vivo* contexts, indicating promising applications in the development of functional foods and nutritional supplements. While the study confirmed the antioxidant activity of glycosylation products using the zebrafish model, zebrafish, as a lower vertebrate, possess a physiological environment that markedly differs from that of humans, potentially limiting their ability to accurately replicate the intricate metabolic pathways or prolonged antioxidant effects observed in humans. Future research should aim to validate these findings using mammalian models.

## Data Availability

The datasets presented in this study can be found in online repositories. The names of the repository/repositories and accession number(s) can be found in the article/supplementary material.
